# From Spin to Swindle: Identifying Falsification in Financial Text

**DOI:** 10.1007/s12559-016-9413-9

**Published:** 2016-05-21

**Authors:** Saliha Minhas, Amir Hussain

**Affiliations:** University of Stirling, Stirling, UK

**Keywords:** Classification, Coh–Metrix, Deception, Financial statement fraud

## Abstract

Despite legislative attempts to curtail financial statement fraud, it continues unabated. This study makes a renewed attempt to aid in detecting this misconduct using linguistic analysis with data mining on narrative sections of annual reports/10-K form. Different from the features used in similar research, this paper extracts three distinct sets of features from a newly constructed corpus of narratives (408 annual reports/10-K, 6.5 million words) from fraud and non-fraud firms. Separately each of these three sets of features is put through a suite of classification algorithms, to determine classifier performance in this binary fraud/non-fraud discrimination task. From the results produced, there is a clear indication that the language deployed by management engaged in wilful falsification of firm performance is discernibly different from truth-tellers. For the first time, this new interdisciplinary research extracts features for readability at a much deeper level, attempts to draw out collocations using *n*-grams and measures tone using appropriate financial dictionaries. This linguistic analysis with machine learning-driven data mining approach to fraud detection could be used by auditors in assessing financial reporting of firms and early detection of possible misdemeanours.

## Introduction

If accounting scandals no longer dominate headlines as they did when Enron and WorldCom imploded in 2001–2002, that is not because they have vanished but because they have become routine

The Economist, Dec 13th, 2014 [1]

Financial statement fraud (FSF) or “book cooking” is a: “deliberate misrepresentation of financial statement data for the purpose of misleading the reader and creating a false impression of an organization’s financial strength” [2]. The deliberate misrepresentation, as outlined in accounting and auditing enforcement releases (AAER) filed by the securities exchange commission (SEC) include improper revenue recognition (the most common), manipulating expenses, capitalizing costs and overstating assets. This type of fraud causes the biggest loss: “a median loss of $1 million per case” [3]. The resultant loss of trust in capital markets and “confidence in the quality, reliability and transparency of financial information” [2] has disastrous implications for jobs, savings and investments. All can be wiped out. The financial industry’s meltdown in 2008 is a perfect example of what catastrophe follows when investors lose trust and confidence.

Financial fraud detection using data mining is a well-developed field. Ngai et al. [4] review the data mining techniques applied in this domain which include categories such as credit card fraud, money laundering and corporate fraud. Results from empirical research indicate successful outcomes from predictive modelling processes [5–7]. The vast majority of this research is based on extracting numerical features (mostly ratios) from financial statements. However, such ratios have limited ability on their own to detect bias/fraud (too many false positives—non-fraudulent document classified as fraudulent) and false negatives (fraudulent document classified as non-fraudulent) [8].

In comparison, there is paucity of analysis on using text as predictors to data mining techniques for financial fraud detection. This is surprising given the increased content of unstructured data with a concomitant increase in language that can be mangled with deceit. Using the financial reporting domain as a microcosm, it is demonstrated in this study that the: “linguistic correlates of deception” [9] could be prized out using techniques demonstrated in this paper.

In the midst of a: “dense and complex web of stakeholder communication” [6] by firms, the annual report/10-K has remained the definitive guide for assessing company health. Again, much of this assessment has been performed using quantitative data in these reports, and the narrative sections have not been so intensely scrutinized. Studies have shown that these narratives provide value-relevant information with respect to a company’s future prospects [10]. By definition, financial statements such as profit and loss, balance sheets, cash flow are retrospective in providing an account of company performance, whereas the narratives are more forward looking with information regarding strategy, business model, risk and uncertainty [11]—all crucial insights needed by potential investors. Therefore, the narrative sections of annual reports/10-K are chosen to test the proposition that fraud firms who have the audacity to misstate numbers would also provide misleading narratives. The 10-K is similar to the annual report but has a distinct structure which is used to convey details on company operations and performance.

There exists substantial evidence that indicates how our choice of words can reveal our inner intentions [12–14]: “a great deal can be learnt about people’s underlying thoughts, emotions, and motives by counting and categorizing the words they use to communicate” [13]. Newman et al. [13] examined a number of narratives from a variety of sources and concluded that the language we use is like a “fingerprint” thus enabling identification of the true meaning behind the words we deploy. From this premise, deception detection research has derived linguistic cues to be found in written text that can aid in separating liars from truth-tellers [14]. Some of these cues are outlined in Table 1 where column 2 illustrates how these cues could be manifested in text with columns 3 and 4 giving reference to the authors and the underlying theories, respectively. Zhou [14] formalised these cues into nine constructs that have been used to automate deception detection with successful outcomes [12, 15].

**Table 1:** Linguistic cues to deception

Deceptive linguistic cues	The effect in text	Author	Theory/method
Word quantity	Could be higher or lower in deceptive text. Generally, higher quantities of verbs, nouns, modifiers and group references	Zhou [14]	Interpersonal deception theory
Pronoun use	First person singular pronouns less frequent, greater use of third person pronouns. This is known as distancing strategies (reducing ownership of a statement)	Newman et al. [13] Zhou [14]	Interpersonal deception theory
Emotion words	Slightly more negativity, greater emotional expressiveness	Newman et al. [13]	Leakage theory
Markers of cognitive complexity	Fewer exclusive terms (e.g. but, except), negations (e.g. no, never) and causation words (e.g. because, effect) and motion verbs—all require a deceiver to be more specific and precise. Repetitive phrasing and less diverse language is more marked in the language of liars. Also, more mention of cognitive operations such as thinking, admitting, hoping	Newman et al. [13] Hancock et al. [12]	Reality monitoring
Modal verbs	Verbs such as would, should and could lower the level of commitment to facts	Hancock et al. [12]	Interpersonal deception theory
Verbal non-immediacy	“Any indication through lexical choices, syntax and phraseology of separation, non-identity, attenuation of directness, or change in the intensity of interaction between the communicator and his referents”. Results in the use of more informal, non-immediate language	Zhou [14]	Interpersonal deception theory
Uncertainty	“Impenetrable sentence structures (syntactic ambiguity) or use of evasive and ambiguous language that introduces uncertainty (semantic ambiguity). Modifiers, modal verbs (e.g. should, could) and generalizing or “allness” terms (e.g. “everybody”) increases uncertainty”	Zhou [14]	Interpersonal deception theory
Half-truths and equivocations	Increased inclusion of adjectives and adverbs that qualify the meaning in statements. Sentences less cohesive and coherent thereby reducing readability	McNamara et al. [18] Bloomfield [29]	Management obfuscation hypothesis
Passive voice	Increase in use, another distancing strategy—switch subject/object around	Duran et al. [50]	Interpersonal deception theory
Relevance manipulations	Irrelevant details	Duran et al. [50] Bloomfield [29]	Management obfuscation hypothesis
Sense-based words	Increase use of words such as see, touch, listen	Hancock et al. [12]	Reality monitoring

Human ability to detect deception is only slightly better than chance (55–58 % range with professional lie catchers only slightly better) [16]. Assessing: “risk is a non-intuitive, humanly biased, cognitively difficult task” [17]. Therefore: “tools that augment human deception detection thereby increasing detection accuracy would prove to be quite valuable” [17].

Given this evidence that there is a linguistic signature to deception and that FSF is an immensely damaging form of deception that needs to be tackled. This study is an endeavour to determine what linguistic constructs would aid in its identification from narrative disclosures in 10-K/annual reports alone.

A prominent data mining technique—classification is rolled out to aid in discriminating narratives of fraud firms from those of non-fraud firms. Three mutually exclusive set of predictors (features) were input into a set of classification algorithms to determine the greatest accuracy. The three sets of predictors wrapped into three distinct approaches are as follows:

- The Coh–Metrix tool was used to extract 110 indices that measure how words are arranged and structured in discourse [18]. Together these indices provide a more robust measure of text readability [18]. Currently, a single measure such as the Gunning fog index has been the de facto standard in disclosure research in determining the readability of financial text [19].
- Multi-word expressions are extracted (bigram and trigrams, known as *n*-grams) from the corpus. Both sets of *n*-grams would pick up greater context and thereby prize out collocations and differences in their use. These linguistic features are strong markers of style, thus would enable detection of any pattern differences.
- Emotionally toned words are often touted as differentiating markers of linguistic style. The huge body of opinion mining and sentiment analysis research focuses on positive/negative polarities of words to gauge intent [20]. In the financial domain, Loughran and McDonald [21] discounted the use of general-purpose dictionaries to detect sentiment in financial text as often: “a liability is not a liability” [21] in this setting. They developed word lists for positive, negative, modal words (weak and strong), passive and uncertainty words. Their view is that these word lists are better suited to a financial setting. In this study, these word lists are used to pick up a frequency count of the words in the lists that are present in the corpus. A word list for forward-looking words was also used. This word list is integral to the WMatrix tool (described below) that is used to interrogate financial narratives. Forward-looking statements have been examined as markers of “informativeness” in financial text [22].

The research question addressed rests on the premise that language deployed by truth-tellers and liars is distinct and can be distilled.

Therefore, is it possible using NLP techniques (classification with a unique set of predictors as described above) to discriminate between narratives of fraud and non-fraud firms?

As far as can be determined to date, this is the first study that uses these predictors for this classification task. Previous studies have examined readability in financial text in a much more limited way using only single scores such as Gunning fog index [22]. Here, 110 indices are extracted that probe the text at a much deeper level for readability. Similarly, there is a paucity of research that examines text for deception using multi-word expression and tonal words designed specifically for the financial domain.

The structure of the paper is as follows: in Sects. 2 and 3 underlying theories in financial reporting and deception are examined, followed by literature review in Sect. 4. Section 5 delineates the methodology. Section 6 relays the results with a discussion. The paper closes in Sect. 7 with a conclusion.

## Financial Reporting

The effects of agency and information asymmetry [23] permeate through all aspects of financial dealing [24]. In financial reporting, two competing explanations are put forth as to how it impinges upon this domain. The first being the efficient market hypothesis view that economic agents act in a rational, utility maximizing manner. The assumption using agency theory is that managers are motivated by incentives to provide: “information gain” [25] as it enhances their reputation and compensation. Investors would then absorb all such data into their rational decision-making process. As a consequence of this rationality and drive to provide value-add information, the incentives for biased reporting is reduced as users driven by utility maximization are able to detect bias.

The other view rooted in behavioural finance theory is that information asymmetry and agency can result in impression management—where managers have the potential to: “distort readers’ perceptions of corporate achievements” [26] by means of obfuscating failures and emphasizing successes [26]. This opportunistic behaviour by managers exploits information asymmetries by releasing biased information as: “cognitive constraints render investors unable to undo reporting bias, resulting in short-term capital misallocations” [27]. This underlying theory directs this research as managers can exploit information asymmetries which can result in bias to falsification in their financial reporting. Management that engage in impression management are not being untruthful but often introduce bias in their narratives to deflect blame for poor performance [2]. Therefore, if the avenue for bias as afforded by agency and information asymmetry is available then the door is open for those who take the leap further into outright falsification. This gives rise to “opportunity” [2] one of the three factors in the fraud triangle as depicted by Rezzae [2]. These factors when present in a firm increase the likelihood of financial statement fraud. The other factor being pressure or incentives for example to meet analyst expectations or high debt. The third is an attitude or rationalization that justifies the misconduct by the perpetrators.

## Language and Deception

How deception manifests itself in language has been wrapped into four approaches: criteria-based content analysis (CBCA), reality monitoring (RM), scientific content analysis (SCAN), verbal Immediacy (VI) and interpersonal deception theory (IDT) (see [15] for elaboration). These theories have derived cues indicative of deception that are manifested in language, see Table 1. For example, according to RM description of events that happened as opposed to falsified events will contain more perceptual and contextual information. CBCA holds that: “truthful messages will contain more unusual details, more superfluous details, more details overall, and more references to time, space, and feelings than deceptive messages” [15]. Therefore, it can be said that deception leaves its mark on language and the challenge is in the detection.

In this study, automated deception detection is attempted in three ways as explained below.

- Syntactical complexity that is readability of accounting narratives have been explored in the literature and used as a device to obfuscate bad news [27, 28]. This is in line with the incomplete revelation hypothesis which maintains that information that is difficult to extract is not impounded into share prices [29].

Traditionally, readability of text has been measured by formulas such as Flesch–Kincaid and Gunning fog. These metrics of text complexity have been found to be highly correlated (*r* > 0.90) [18]. This correlation exists because most readability measures in use include features related to the frequency of a word in language and the length of the sentence [18]. These measures have been discounted [18] as they: “ignore many language and discourse features that are theoretically influential at estimating comprehension” [18].

Further, Loughran and McDonald [21] also cast doubt on the suitability of these traditional metrics to ascertain the readability of financial text. They argue that multisyllabic words in 10-K filings are dominated by common business words that should be easily understood. They maintain that words like “company”, “operations” and “management” will not confuse readers of SEC filings. However, using metrics such as Flesch–Kincaid or Gunning fog would result in an incorrect score on readability.

**Fig. 1:**
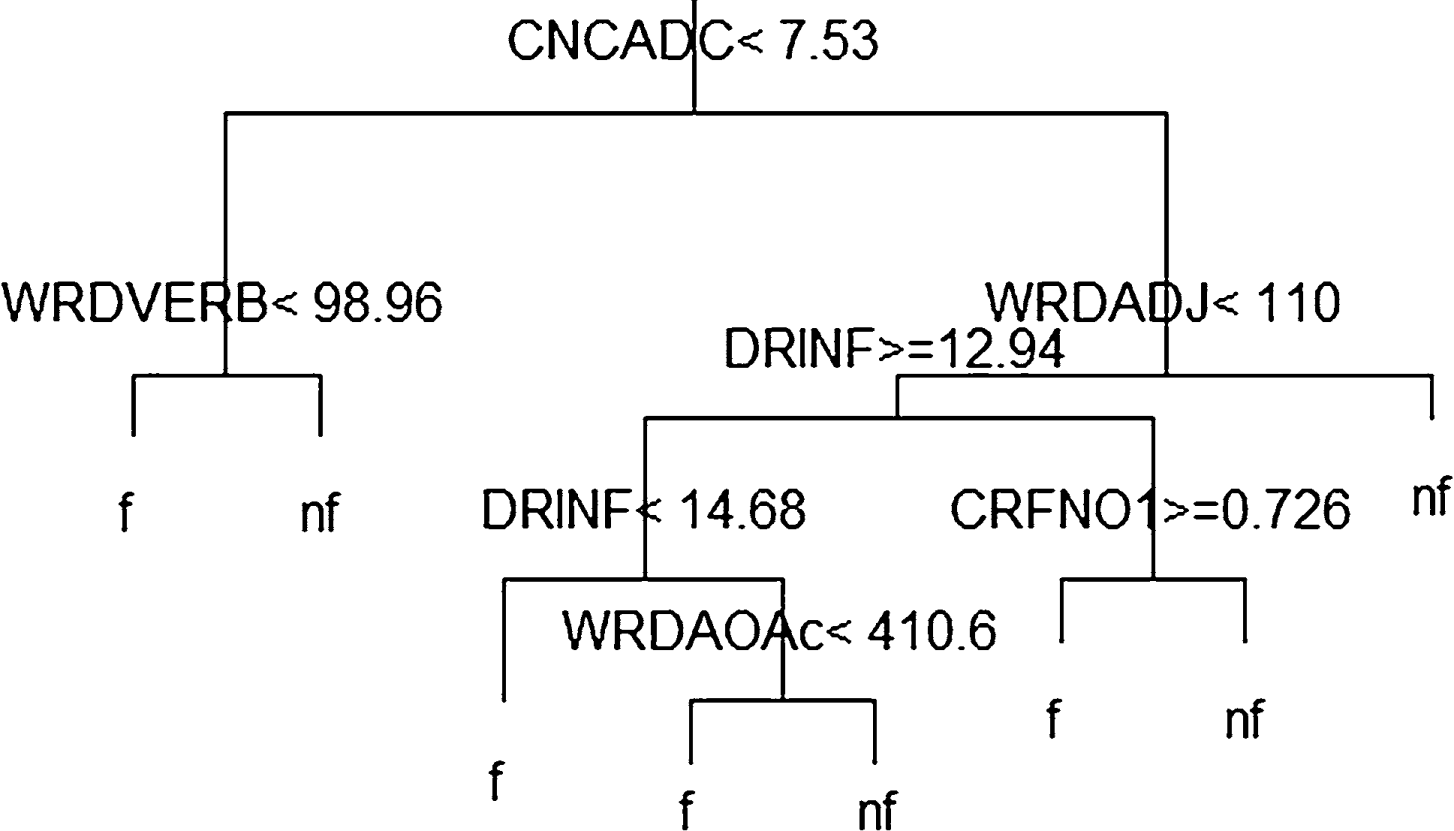
A section of a decision tree

To correct this deficiency and to take a more rigorous measure of readability, Coh–Metrix is used in this study. This is a robust natural language processing (NLP) tool that advances the readability measure by embracing cohesiveness and cohesion in text. This is the: “linguistic glue that holds together the events and concepts conveyed within a text” [18]. Once Coh–Metrix is executed over the text, it produces 110 indices. Figure 1 shows an extract of indices being used to determine differences in their use between fraud and non fraud firms.

These indices give a score for measures such as referential and semantic overlap of adjacent sentences, number of connectives and a word concreteness score (words that are easy/difficult to process). Cohesive cues enable the reader to make connections between sentences and paragraphs. This is measured for example by calculating overlapping verbs and connectives (causal, intentional, temporal). Other indices measure aspects of text such as referential overlap, latent semantic similarity, narrativity (the degree to which a text tells a story with characters, events, places and things that are familiar to a reader). McNamara et al. [18] give a full explanation of these indices and how they are calculated. The tools used to calculate the indices include: “lexicons, syntactic parsers, part of speech classifiers, semantic analysis, and other advanced tools in NLP” [12].

- Sinclair [30] maintains that language is 70 % formulaic, and there is less variability in its use that would be garnered from the term popularised by Chomsky [31] that language is: “infinite use of finite means” [31]. This would be especially true when examining a particular genre of text such as financial text, where content, structure of discourse and linguistic style would be similar. Therefore, any difference in key constructs of language like collocations could be significant. In this study, multi-word expressions such as bigrams and trigrams are picked up from the corpus to aid in fraud detection.
- The tone in financial text has been investigated to aid in determining company intentions and to predict stock price movement [28, 32]. The tone in text has primarily been gauged using general-purpose dictionaries used like Diction, Harvard Psychosociological dictionary [33]. Loughran and McDonald [21] find that these dictionaries substantially misclassify words in determining tone in financial text. They create a positive and a negative word list that are more appropriate. They also devised word list that relates to certainty, passive, modal strong and weak words. As can be seen from Table 1 that shows possible linguistic markers of deception, these words could aid in discriminating a fraud from a non-fraud firm.

Further, there is body of literature [22, 34]) that emphasizes the informativeness associated with temporal components such as forward-looking words in interpreting financial narratives. Therefore, a forward-looking word list and Loughran and McDonald [21] word list are deployed to aid in the discrimination task.

## Literature Review

Numerous studies have been conducted in the area of financial fraud predictions from quantitative data [35, 8, 36]. For example, Ngai et al. [4] present a review of all the data mining techniques used to aid financial fraud detection and categorise all the different types of financial fraud that exist. Their findings indicate that classification and clustering are the data mining techniques of choice for the different types of financial fraud that exist. Similarly, Sabau et al. [37] and Chintalapati et al. [35] provide similar reviews on the use of data mining techniques to detect financial fraud. Ravisankar et al. [7] find that probabilistic neural network and genetic programming give the best results using financial ratios to detect fraud. Perols [6] finds that support vector machine and logistic regression classifiers perform well relative to others on again using quantitative data from financial statement as predictors to FSF detection.

In contrast, studies on financial narratives are less numerous. Merkl-Davies and Brennan [27] provide a comprehensive literature review of manual/semi-automated content analysis approaches on narrative sections of corporate reports. They uncovered seven main strategies that can be deployed in narratives for impression management. The strategies pertinent to this research are:

- Obfuscating bad news through reading ease or rhetorical manipulation. The motivation is that managers make the text less clear so that information is more costly to extract and poor performance will not be reflected immediately in market prices. Similarly, the use of rhetorical language deployed through the use of pronouns, passive voice, metaphor has been used to conceal poor firm performance. They argue that it is not: “what firms say” but rather “how they say it” [27] that leads to obfuscation. This is known as management obfuscation hypothesis. Most of the studies in this area use the Flesch–Kincaid or Gunning fog score to measure readability or manual content analysis to pick up rhetorical language constructs [28].
- Emphasising good news through thematic manipulation, this is the “pollyanna principle” at work where managers emphasize good news and conceal bad news. This would result in greater positive overtones. In an management discussion and analysis (MDA) section of annual reports, this would manifest as: “presenting a false version of past performance, an unrealistic outlook for the future, misrepresenting the significance of key events omitting significant facts, providing misleading information about the current health of the company” [26]. To date, the tone in financial text has been examined using manual/semi-automated content analysis techniques based on positive/negative word counts [27].

The tone used in financial narratives has been shown to impact the stock market. For example, Tetlock [38] shows that pessimism puts a downward pressure on prices of stock indices. They used Loughran and McDonald’s word lists to measure tone. Feldman [28] study reveals markets react to positive/negative tone in MDA sections of annual reports. Kothari et al. [33] found that tone determines how financial narratives are interpreted as favourable/unfavourable which in turn affects the firms risk stature. In these two studies, tone was picked up using general-purpose dictionaries. All of this work is based on semi-automated/manual content analysis.

Li [19] conducted a seminal study on the relationship between readability of annual reports and financial performance. He found that a higher reading of the fog index indicates disclosures that are more difficult to understand. He finds a negative relationship between fog and the level of earnings. A recent study, using 216 disclosures by fraudulent firms by Kin et al. [39] confirms that readability has incremental power in predicting financial misstatements. However, again readability was measured using only the Gunning fox Index.

Cecchini et al. [40] develop a methodology to analyse text to detect fraud and bankruptcy outcomes. They do this by creating a dictionary of terms (an ontology) from annual reports that can be used to discriminate firms that encounter catastrophic financial events. Zhou and Kapoor [41] recommend developing fraud detection mechanisms that are adaptive to the chameleon nature of fraud. They propose a new self-adaptive framework that incorporates domain-specific knowledge along with the inclusion of variables that predispose companies to fraudulent activity, uncovered by Rezaae [2].

Humphreys et al. [15] used Zhou et al. [14] linguistic-based cues formulated into ratios to differentiate between fraud and non-fraud firms. They find that the former use more: “activation language, words, imagery, less lexical diversity”. These ratios form the feature set in a suite of classification algorithms. This study is the most similar to this one. However, the attempt here is to probe the text deeper, using more robust NLP technology (Coh–Metrix) to pick up more language features in the text. Goel et al. [42] train a support vector machine (SVM)-based learning algorithm using surface language features deemed to be indicative of fraud such as the percentage of passive voice sentences. Using an SVM classifier, they achieved 89 % classification accuracy. Glancy and Yadav [43] test for fraud in financial reporting by constructing a term-document matrix pulling out terms from MDA sections of annual reports using companies that have been indicted for fraud. They then apply singular-value decomposition to improve distinction between fraud and non-fraud firms.

Purda and Skillicorn [44] develop a tool for distinguishing between fraudulent and truthful reports based on the language used in the MDA section of annual reports. The data are used to identify words most strongly associated with financial misrepresentation. This is in contrast to using predefined word list to determine associations between words and type of reports (fraud or non-fraud). They find that their data-driven approach has a superior classification performance.

This study contributes to the above body of work by seeking to show that readability measures need to be advanced. Coh–Metrix is a tool that can lead the way forward. Its efficacy has been tested using a newly developed corpus of narratives of fraud/non-fraud firms. Given that management engaged in deception have been shown to reduce readability in their financial disclosures, Coh–Metrix would be ideally suited to measure this construct in this context. A data generated approach is also attempted here but not using unigrams for bag of words as undertaken by Purda and Skillicorn [44] but using bigrams and trigrams. This would allow a greater capture of context and thus could aid in the discrimination task. A more replete financial word list is also used in this study. It is more replete than previous work as it not only uses appropriate financial dictionaries but also uses a word list that contains forward-looking words. This could aid in highlighting differences in the “informativeness” between the two types of reports in the corpus and thus assist in the discrimination task.

## Methodology

### Data and Tools

The corpus consists of 51 fraud firms and 153 non-fraud firms between 1989 and 2012. A wide time period enabled a better catchment of prominent fraud cases (Worldcom, Enron, Tyco). The fraud set was primarily gathered by interrogating accounting and auditing enforcement releases (AAERs) from the securities exchange commission (SEC) website (www.sec.gov). Only those firms that had materially misstated their 10-K reports were selected. The 10-K’s for the year of the fraud were retrieved from the SEC EDGAR database. The 10-K 1 year prior to the fraud was also collected and added to the corpus. Therefore, for each fraud firm, there were two 10-K’s (the fraud year and the fraud year −1). “Book cooking” usually has been simmering for a few years prior to discovery [32], and this approach picks up more firm narratives that are falsified. The sections of the 10-K that were added to the corpus in text files were Item 1 Business, Item 7 Management Discussion and Analysis of Results of Operations and Financial Condition, and Item 7A Quantitative and Qualitative Disclosures about Risk. These sections are most likely to contain the narratives that deal with risk and uncertainty, business model and strategy where falsification is most likely to occur. For firms with no 10-K, extracts were taken from their annual report. Sections from the narratives sections excluded were corporate governance, notes to financial statement and any sections on corporate social responsibility.

In keeping with previous research in this area of falsification in narrative sections of 10-K’s/annual report [15, 40, 42] each fraud firm was matched in terms of size, industrial grouping and operating profit with three non-fraud firms. The screening was performed to determine the non-fraudulent status of the firm using market reports, competitor profiles and financial statement assessment.

Both a peer set for each fraud company and a matched-pair data set scenario were established.

In the former case, the set-up is that for each fraud firm represented by two annual reports/10-K is matched with three non-fraud firms each represented by two annual reports/10-K. This gives a ratio of two fraud reports to six non-fraud reports resulting in 102 fraud reports to 306 non-fraud reports giving a total of 408 reports. The aim is to show that the features chosen as discriminators are prominent enough to indicate a fraud firm, despite the greater non-fraud reports. It is also likely that it mimics a more true to life portrayal with non-fraud firms outnumbering fraud firms. This set-up was also the preferred method by Goel et al. [42]. They also cited a possible realistic reflection of the prevalence of fraud for this choice.

In the other case of one fraud firm to one non-fraud firm results in two fraud reports and two non-fraud reports. This produces a corpus of 204 reports (102 fraud to 102 non-fraud) giving a balanced data set. Now, a direct comparison between the two data sets can be made. As in some cases with an unbalanced data set, the learning algorithms are more predisposed to labelling all cases with the majority class (non-fraud) [45]. The first set-up described above will be referred to as the peer set scenario and the other one as matched-pair scenario.

All formatting was removed from all extractions and pasted into text files. The following tools were used to perform the linguistic analysis.

### Text Mining (tm) Package in R

Transform the texts into structured representations where existing R methods can be applied, for example, clustering and classification [46].

### Caret Package in R

This is a set of functions that attempt to streamline the process for creating predictive models [47].

### WMatrix-Import Web Tool

Permits large batch scoring of 10-K/annual reports [48]. The word lists and the reports are imported into the tool. Loughran and McDonald’s [21] word lists (positive, negative, modal strong, modal weak, uncertainty, passive) were used. A word list associated with forward-looking words (97 words) was also used. This word list was devised by Hajj et al. [48]. The tool reads the word lists and scans each report for words that occur in the word list and in the report. It outputs a total count of words in word lists found in the report.

### Boruta Package in R

This is a feature selection—random forest algorithm. It removes features which are proved by a statistical test to be less relevant. The importance of each variable is given by a *z* score and is compared to a random permutation of the variables to test if it is higher than the scores from random variables [49]. This step also reduces statistical overfitting which causes small and largely irrelevant differences between deceptive and truthful conditions to be exaggerated [50]. Further, the dimensionality of the data is reduced and the classification algorithms run faster.

### Feature Extraction

As already indicated, the first set of features is the Coh–Metrix indices. Before the text could be passed to Coh–Metrix, it required extensive cleaning as outlined in [18]. Simply put the text had to look as if: “the writer had just finished typing it, had it checked for typos and errors by a large group of copy editors, printed if off, and handed it over to the reader” [18].

**Fig. 2:**
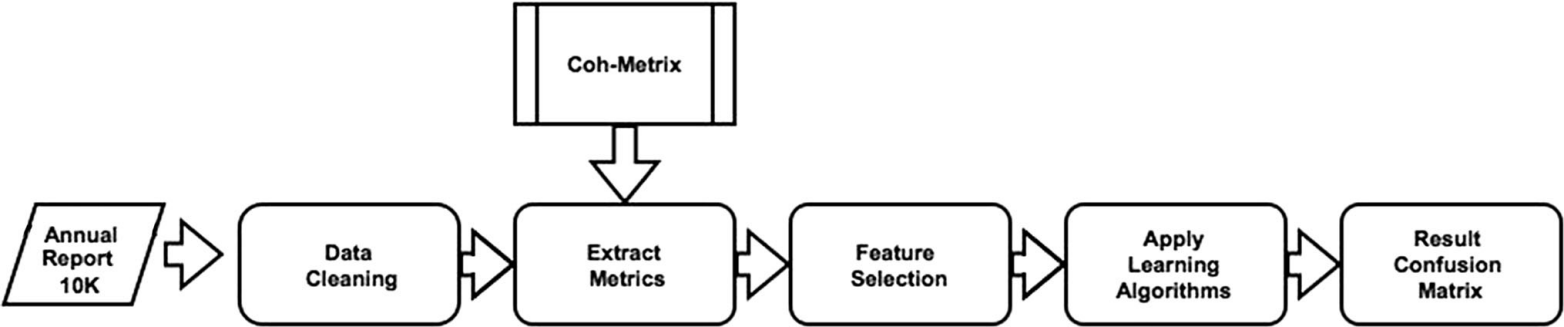
Approach 1, feature extraction using Coh–Metrix Indices

**Fig. 3:**
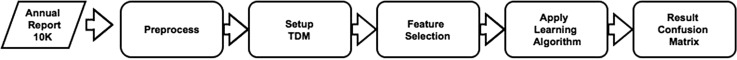
Approach 2, feature extraction using multi-word expressions

This first approach to classification—approach one—using the Coh–Metrix indices is shown in Fig. 2. Once the text was cleaned, the Boruta feature selection picked the most prominent indices. These were then used as features to represent the reports of fraud firm and non-fraud firms. Approach two which involved the extraction of bigram/trigram (*n*-grams) counts is shown in Fig. 3. In this approach, the reports (fraud/non-fraud) were put through a preprocessing routine in text miner. This involved, converting text to lowercase, white space, number and sparse term removal. A term-document matrix (tdm) was then generated. The columns are the bigram or the trigrams found in the text, with the cells being the frequency counts. These bigrams/trigrams were also put through Boruta feature selection to pick up the most significant *n*-grams.

**Fig. 4:**
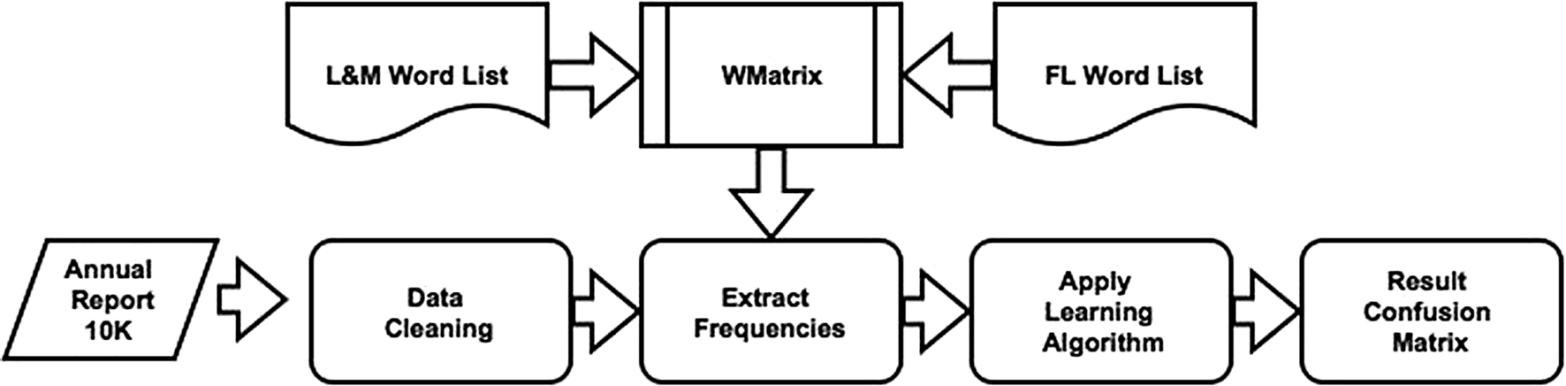
Approach 3, using tonal words

Approach three shown in Fig. 4 involved set-up of custom dictionaries in WMatrix. Loughran and McDonald [21] derived 5 word lists from an extensive sample of 10-K reports. The words in these word list they argue better reflect tone in financial text. The word list contain words that relate to positive, negative sentiment, both strong and weak modal words, passive verbs and words that indicate uncertainty. Outwith Loughran and Mcdonald’s word list another word list based on words that denote future intent (forward looking) was added. This word list was devised by Hajj et al. [27] based on a study into annual reports produced by UK based firms. The frequency count from each of these word lists constitute the seven features used in this approach. The frequency is given as a proportion of total words in the document. These features are then passed to the classification algorithms.

### The Classification Task

The classification task in this study was performed using supervised machine learning techniques. This involved: “learning a set of rules from instances (examples in a training set), or more, generally speaking, creating a classifier that can be used to generalize from new instances [51]”.

The text classification task is a mapping process. Text is assigned to one or more classes from a predefined set of classes (fraud (*f*) or non-fraud (*nf*)), and this can be done in different ways [51].

- *x* = is the firm narratives
- *y* = {*f*, *nf*} of the possible classes

Classifier *Y* maps input to class

$$ Y:x \to y $$

Goal: Determine the function for this classifier with a training set D composed of *n* examples

1 $$ \left\{ {\left( {x,y} \right): \le i \le n} \right\} $$

The goal is to use this data to obtain an estimate of the function that maps the input vector *x* into the values of the class *y* (*f, nf*). This function can then be used to make predictions in instances where only the values are observed. This prediction or target function is also known as a classification model. Formally: “a prediction function should learn that $\hat{f}\left( X \right):x \to y$ minimises the expectation of some loss function *L*(*y*, *f*) over the joint distribution of all (*y*, *x*) values” [52].

2 $$ \hat{f} \left( x \right) = \arg \min_{f\left( x \right)} E_{y,x} L\left( {y,f\left( x \right)} \right) $$

A classifier is an approach used to build classification models. Examples include decision tree classifiers and support vector machines that are used in this study. As indicated a learning algorithm is employed to identify a model that best fits the relationship between the attributes (the features from the three approaches) and the class label (fraud or non-fraud). The model generated by a learning algorithm should fit both the input data well and correctly predict class labels of reports it has never seen before.

### Decision Tree Classifiers

These classifiers pose a series of carefully crafted questions about the attributes of the test record. Each time an answer is given, a follow-up question is asked until a conclusion about the class label of the record is reached. These questions and answers are in the form of a tree structure. The root and internal nodes contain attribute test conditions to separate records that have different characteristics. All terminal nodes are assigned a class label fraud or non-fraud. Once the decision tree is constructed, classifying a test record should be as easy as answering the questions that are intertwined in the tree and going down the appropriate branch until the class label is reached. The decision tree inducing algorithm must provide a method for specifying the test condition for different attribute types as well as an objective measure for evaluating the goodness of each test condition. This is usually performed using purity/impurity measures such as Gini Index, entropy and misclassification error [53].

An example of a sub-region of a tree generated from the corpus using the Coh–Metrix features is shown in Fig. 1.

### Random Forests and C5

These are decision trees that improve predictive accuracy by generating a large number of bootstrapped trees (based on random samples of variables), classifying a case using each tree in this new “forest”, and deciding a final predicted outcome by combining the results across all of the trees (a majority vote in classification).

The C5 classifier is also an extension of the basic decision tree approach. It constructs a decision tree to maximize information gain (entropy). At each node, the attribute with the highest normalized information gain is chosen to make the decision that most effectively splits the samples into subsets.

### Stochastic Gradient Boosting (SGB)

This classifier computes a sequence of very simple trees. Each successive tree is built for the prediction residuals of the preceding tree. The model assumes an additive expansion:

3 $$ F\left( {x,\beta ,\alpha } \right) = \mathop \sum \limits_{i = 0}^{n} \beta_{i} h\left( {x,\alpha_{i} } \right) $$

The ‘*h*’ are the weak learners. The predictor from gradient boosting is a linear combination of weak learners and the procedure does two things:

- Computes *β*_*m*_—the weight of a given classifier.
- Weights the training examples to compute the *i*th weak classifier *h*(*α*_*m*_).

The final classifier is a weighted majority vote of the individual weak classifiers [52]. The weak learners are a method of converting rough rules of thumb into highly accurate prediction rule.

The full algorithm with pseudo code is delineated in Friedman’s seminal paper [54] on this model.

### Support Vector Machine (SVM)

The support vector machine (SVM) classifier finds a hyperplane that can separate *f/nf* classes and has the largest distance between borderline f/nf cases (i.e. the support vectors). Therefore:

4 $$ \vec{w} \cdot \vec{x}_{i} + b \le - 1\quad {\text{if}}\quad y_{i} = - 1 $$

5 $$ \vec{w} \cdot \vec{x}_{i} + b \ge + 1\quad {\text{if}}\quad y_{i} = + 1 $$

6 $$ \vec{w} . \vec{x}_{i} + b =      0 $$

Equation (6) represents the hyperplane, whilst Eqs. (4) and (5) represents the non-fraud instances and fraud instances, respectively. The SVM model is then an optimization problem. Minimise an objective function, subject to the above constraints as shown in Eqs. (4) and (5).

### Boosted Logistic Regression

The other classifier used is boosted logistic regression. This attempts to separate the classes (fraud and non-fraud) along a sigmoid function (*s*-shaped) are denoted by:

7 $$ 1/\left( {1 + {\text{e}}^{{\theta_{x}^{\text{T}} }}  } \right) $$

Once the reports have been separated using the function, the following equality check is performed:

- If *h*_*θ*_(*x*) ≥ 0.5 predict fraud. If *h*_*θ*_(*x*) < 0.5 predict non-fraud.

A prime test on the efficacy of these machine learning classification models is the amount of bias and variance that exists in the model. Bias or underfitting is when the model does not capture the general pattern in the data, whereas variance or overfitting is the opposite where model matches idiosyncrasies in the data. The fraud classification task under study has a complex, nonlinear boundary. This can result in high bias when using linear classifiers such as logistic regression. Nonlinear classifiers such as SVM, SGB and the decision trees can be performed better but have variable performance as they are more predisposed to variance. Both the SVM and SGB models are better able to handle unbalanced data sets, as can be deduced from previous studies [55, 56]. SVMs are also better able to handle high dimensionality that is characteristic of the feature vectors in this study [8].

For the classification task, 75 % of the data was used for training and building the classifier. The remaining 25 % was used to test the accuracy of the classifier. The training set is used: “to estimate model parameters” [57], whilst the test set is: “used to get an independent assessment of model efficacy” [51]. The test set is not used during model training. Once the tuning parameters for a model are set, the resampling methods are specified. In all models, the widely used repeated *k*-fold cross validation is deployed (three separate 10-fold cross validations is set). This enables, in the absence of a large test set, an estimate of the test set prediction error. The data are: “divided into K equal parts, one *K* part is left out, the model is fitted to the other *K* − 1 parts (combined) and predictions obtained for the left out the *k*th part, this is done for each part” [51]. An overall accuracy estimate is provided. This approach shakes ups the data; however, each *k* is only as big as the original training set and prediction error could be biased upwards.

Feature extraction for the three approaches is further delineated below. There were no real metrics that were necessary for feature extraction. For approach 1, all the 110 indices given by Coh–Metrix were considered for use as features. Similarly for approach 2, all *n*-grams generated were considered. However, the Boruta feature selection algorithm reduced these features for both approaches 1 and 2. This algorithm is known as an all relevant feature selection method. This means it tries to find all features carrying information usable for prediction. It does this by: “comparing original attributes’ importance with importance achievable at random, estimated using their permuted copies” [49]. For approach 3 raw counts of words in corpus that were in the seven word lists were all extracted for input to classifiers. No feature selection was necessary for this approach.

Five models from that provided the highest classification accuracies were tabulated.

## Results and Discussion

### The Three Approaches

A battery of measures are taken to provide a comprehensive outlook on classifier performance. The results for these metrics attained by the classifiers for the feature sets used are all shown in Tables 4, 5, 6 and 7. The best overall performing classifiers in both the peer set and matched-pair data sets are highlighted in bold. The results shown are generated from the confusion matrix command in R. A basic definition of these metrics is delineated below.

### Kappa

A metric that compares observed accuracy with expected accuracy (random chance). Therefore, a measure of prediction performance of classifiers.

### Accuracy (ACC)

The number of correct predictions from all predictions made.

### Sensitivity (True Positives)

The proportion of fraud reports, correctly identified.

### Specificity (True Negatives)

The proportion of non-fraud reports, correctly identified.

### No Information Rate (NIR)

Largest proportion of the observed classes. In the peer set scenario, there were more non-fraud reports than fraud reports in the corpus and therefore more non-fraud in the test cases, whereas in the matched-pair design, there were equal numbers of fraud and non-fraud reports.

### *P* Value (ACC > NIR)

A hypothesis test is computed to evaluate whether the overall accuracy rate is greater than the rate of the largest class. *P* values lower than 0.05 indicate a significant difference.

### Pos Pred Value (PPV)

The per cent of predicted positives (fraud) are actually positive. In other words, it is the probability that a report designated as fraudulent is truly fraudulent.

### Neg Pred Value (NPV)

The per cent of negative positives (non-fraud) are actually negative. Again, it can be expressed as the probability that a report designated as non-fraudulent is truly non-fraudulent.

### Balanced Accuracy

Arithmetic mean of sensitivity and specificity values.

Given the above definitions, a well-performing classifier would have higher kappa values, higher sensitivity and (PPV) scores as they are complimentary, higher specificity and NPV scores (also complimentary), higher accuracy (ACC) and balanced accuracy score (again complimentary) and low p values (ACC > NIR).

Results are only shown for the top five best-performing classifiers from the caret package. For the peer set scenario, the classifiers were trained on 307 reports. The 101 remaining reports were used as test cases against the trained classifier to predict report class (fraud or non-fraud). For the matched-pair design scenario, 153 reports were used to train the classifier, leaving 51 reports to be used as test cases. The results are shown in Tables 4, 5, 6 and 7. Accuracy is based on the number of test cases whose class designation (fraud or non-fraud) were correctly predicted by the classifier. This prediction is based on the learning functions derived using a training set by the classifiers shown in the tables.

#### Approach One

The standard 110 indices produced by Coh–Metrix were reduced to 29 after the Boruta feature selection algorithm had been executed over the indices.

The indices chosen are shown in Table 2. Indices that begin with:

- “CN” are density scores (occurrence per 1000 words) for different types of connectives. These are important for the: “creation of cohesive links between ideas and clauses” [18].
- “CR” takes measures related to referential cohesion, which refers to the overlap in content words between local sentences.
- “DR” measures syntactic pattern density. All these measures are density scores for grammatical constructs such as noun phrases. This can adversely impact interpretability of text [18].
- “LS” is Latent Semantic Analysis, and this provides a measure of semantic overlap between sentences.
- “PC” these measures provide an: “indication of text-ease or difficulty that emerge from the linguistic characteristics of the text” [18].
- “SM” the strength of mental representation evoked by the text that goes beyond the explicit words.
- “SY” gives measures for how syntactically heavy a sentence is e.g. syntax in text is easier to process when there are shorter sentences.
- “WR” density scores for grammatical constructs. These indices are then input into the classification algorithms. Results are shown in Table 4.

**Table 2:** Coh–Metrix indices chosen by Boruta feature selection

Bigrams	Trigrams
Accounted for	An adverse effect
Acquisition of	And sale of
And sale	At the time
Annual report	Company’s ability to
Be required	During the period
Company in	Entered into a
Continued to	For the year
Designed for	In the event
Due to	May be required
Event that	Million at December
Experience in	Million in cash
For fiscal	Million of cash
Group of	Not believe that
In and	Of our common
In compared	Our common stock
Into a	Primarily as a
Legal and	Primarily due to
Market our	Provided by financing
Necessary to	Pursuant to the
Of approximately	Shares of common
Our management	The acquisition of
Our own	The company in
Purchase price	The company’s ability
The acquisition	The fiscal year
The fiscal	The impact of
The worlds	The results of
To conduct	The year ended
Year ended	Use of the

#### Approach Two

After preprocessing, a large-term document matrix of raw frequency scores was generated for bigrams and trigrams. This matrix was then put through the feature selection programme which output only 28 bigrams and trigrams to be significant discriminators. The selected bigrams and trigrams features are shown in Table 3. Results are shown in Tables 5 and 6, respectively.

**Table 3:** *n*-Grams chosen by Boruta feature selection

Coh–Metrix indices	Description
CNCADC	Density score of adversative/contrastive connectives
CNCAdd	Additive connectives incidence
CNCNeg	Negative connectives incidence
CNCTempx	Adversative and contrastive connectives incidence
CRFANP1	Anaphor overlap, adjacent sentences
CRFNO1	Avg num.(local) sentences that have noun overlap
CRFNOa	Noun overlap of each sentence with every other sentence
CRFSOa	Match of nouns and contents words with common lemma between sentences
DRGERUND	Gerund density, incidence
DRINF	Infinitive density, incidence
DRPVAL	Density score of agentless passive voice form
DRVP	Verb phrase density, incidence
LSASS1d	LSA overlap, adjacent sentences, standard deviation
PCCNCz	Text Easability PC word concreteness, *z* score
PCCONNz	Text Easability PC connectivity, *z* score
PCNARz	Text Easability PC narrativity, *z* score
PCVERBz	Text Easability PC verb cohesion, *z* score
RDFKGL	Flesch–Kincaid grade level
SMCAUSlsa	LSA verb overlap
SMCAUSwn	WordNet verb overlap
SYNLE	Mean number of words before the main verb of the main clause in sentences
SYNSTRUTa	Sentence syntax similarity, adjacent sentences, mean
SYNSTRUTt	Sentence syntax similarity, all combinations, across paragraphs, mean
WRDADJ	Adjective incidence
WRDAOAc	Age of acquisition for content words, mean
WRDFRQa	CELEX Log frequency for all words, mean
WRDIMGc	Imagability for content words, mean
WRDMEAc	Meaningfulness, Colorado norms, content words, mean
WRDVERB	Verb incidence

#### Approach Three

Seven word lists positive, negative, uncertainty, modal words weak, modal words strong, passive verbs, forward-looking (FL) were used in this approach.

Each word list was imported into WMatrix and the result (number of times words in word list found in 10-K/annual reports) added to a matrix.

For the peer set scenario, the final matrix was composed of 408 rows (the reports, fraud and non-fraud) with seven columns (the word lists) with the cells containing the raw frequency counts. For the matched-pair set scenario, the number of rows are reduced to 204 and the same number of columns. The classification results are shown in Table 7.

### Discussion

In the peer set scenario, the corpus is unbalanced. There are three times more non-fraud reports than fraud reports. This ratio is maintained in the training and testing sets. This corpus is set up as such to mimic a possible real-word scenario where there are more non-fraud firms than fraud firms. In such a situation, it is very misleading just to look at classification accuracy as a measure of success. A high classification accuracy could be attained by all non-fraud reports correctly identified with no fraud reports identified, but this would be a model with poor predictive ability. In this case, to ascertain the predictive power of the classifiers, other performance metrics need to be examined, as given in Tables 4, 5, 6 and 7. The best overall performance in approach one, using the Coh–Metrix indices, was achieved by stochastic gradient boosting and the random forest model. These models consistently identify the fraud reports at a higher rate than the others. This is corroborated by the higher kappa scores. The significant sensitivity values indicate that the correct identification of the fraud reports are also contributing to the classification accuracy attained.

**Table 4:** Coh–Metrix

Model	Kappa	Sensitivity	Specificity	ACC	95 % CI	NIR	*P* value [ACC > NIR]	Pos Pred value	Neg Pred value	Balanced accuracy
*Coh–Metrix—peer set scenario*										
Stochastic gradient boosting	**0.63**	**0.68**	**0.94**	**0.88**	**0.80, 0.93**	**0.75**	**0.001**	**0.80**	**0.90**	**0.81**
Boosted classification trees	0.42	0.40	0.96	0.82	0.73, 0.89	0.75	0.06	0.76	0.82	0.68
Support vector machines	0.47	0.40	0.98	0.84	0.75, 0.90	0.75	0.02	0.90	0.83	0.69
C5	0.56	0.56	0.94	0.85	0.46, 0.94	0.75	0.01	0.77	0.86	0.75
Random forest	**0.54**	**0.74**	**0.80**	**0.77**	**0.68, 0.85**	**0.75**	**1.141e−08**	**0.79**	**0.80**	**0.77**
*Coh–Metrix—matched-pair set scenario*										
Stochastic gradient boosting	**0.56**	**0.76**	**0.80**	**0.78**	**0.64, 0.88**	**0.5**	**4.511e−05**	**0.79**	**0.76**	**0.78**
Boosted classification trees	0.36	0.64	0.72	0.68	0.53, 0.80	0.5	0.007	0.69	0.66	0.68
Support vector machines	0.68	1.0	0.68	0.84	0.70, 0.92	0.5	5.818e−07	0.75	1.00	0.84
C5	0.44	0.92	0.52	0.72	0.57, 0.83	0.5	0.001	0.65	0.86	0.72
Random forest	**0.68**	**0.88**	**0.80**	**0.84**	**0.70, 0.92**	**0.5**	**5.818e−07**	**0.81**	**0.86**	**0.84**

Bold values indicate best performing classifiers

**Table 5:** Bigrams

Model	Kappa	Sensitivity	Specificity	ACC	95 % CI	NIR	*P* value [Acc > NIR]	Pos Pred value	Neg Pred value	Balanced accuracy
*Bigrams—peer set scenario*										
Stochastic gradient boosting	**0.60**	**0.56**	**0.97**	**0.87**	**0.79, 0.92**	**0.75**	**0.002**	**0.87**	**0.87**	**0.76**
Random forest	0.58	0.56	0.96	0.86	0.77, 0.92	0.75	0.005	0.82	0.86	0.76
Support vector machines	**0.65**	**0.64**	**0.96**	**0.88**	**0.80, 0.93**	**0.75**	**0.001**	**0.84**	**0.89**	**0.80**
Boosted logistic regression	0.59	0.55	0.97	0.87	0.78, 0.83	0.77	0.01	0.84	0.88	0.76
C5	0.57	0.60	0.93	0.85	0.76, 0.91	0.75	0.01	0.75	0.87	0.76
*Bigrams—matched-pair set scenario*										
Stochastic gradient boosting	**0.52**	**0.76**	**0.76**	**0.76**	**0.61, 0.86**	**0.5**	**0.00015**	**0.76**	**0.76**	**0.76**
Random forest	0.52	0.72	0.80	0.76	0.61, 0.86	0.5	0.00015	0.78	0.74	0.76
Support vector machines	**0.56**	**0.76**	**0.80**	**0.78**	**0.64, 0.88**	**0.5**	**4.511e−05**	**0.76**	**0.79**	**0.78**
Boosted logistic regression	0.52	0.77	0.75	0.76	0.59, 0.88	0.5	0.0023	0.73	0.78	0.76
C5	0.40	0.72	0.68	0.70	0.55, 0.82	0.5	0.0033	0.69	0.70	0.70

Bold values indicate best performing classifiers

**Table 6:** Trigrams

Model	Kappa	Sensitivity	Specificity	ACC	95 % CI	NIR	*P* value [Acc > NIR]	Pos Pred value	Neg Pred value	Balanced accuracy
*Trigrams—peer set scenario*										
Stochastic gradient boosting	0.65	0.76	0.90	0.87	0.79, 0.92	0.75	0.002	0.73	0.92	0.83
Random forest	**0.59**	**0.60**	**0.94**	**0.86**	**0.77, 0.92**	**0.75**	**0.005**	**0.78**	**0.87**	**0.77**
Support vector machines	**0.61**	**0.60**	**0.96**	**0.87**	**0.79, 0.92**	**0.75**	**0.002**	**0.83**	**0.87**	**0.78**
C5	0.62	0.64	0.94	0.87	0.79, 0.92	0.75	0.002	0.80	0.88	0.79
Boosted logistic regression	0.54	0.59	0.92	0.83	0.73, 0.90	0.74	0.02	0.72	0.86	0.75
*Trigrams—matched-pair set scenario*										
Stochastic gradient boosting	0.44	0.72	0.72	0.72	0.57, 0.83	0.5	0.0013	0.72	0.72	0.72
Random forest	**0.68**	**0.96**	**0.72**	**0.84**	**0.70, 0.92**	**0.5**	**5.818e−07**	**0.77**	**0.94**	**0.84**
Support vector machines	**0.60**	**0.88**	**0.72**	**0.80**	**0.66, 0.89**	**0.5**	**1.193e−05**	**0.75**	**0.85**	**0.80**
C5	0.56	0.84	0.72	0.78	0.64, 0.88	0.5	4.511e−05	0.75	0.81	0.78
Boosted logistic regression	0.40	0.85	0.53	0.69	0.54, 0.86	0.5	0.1045	0.73	0.70	0.72

Bold values indicate best performing classifiers

**Table 7:** Financial word lists

Model	Kappa	Sensitivity	Specificity	ACC	95 % CI	NIR	*P* value [Acc > NIR]	Pos Pred value	Neg Pred value	Balanced accuracy
*Financial word lists—peer set scenario*										
Stochastic gradient boosting	0.60	0.56	0.97	0.87	0.79, 0.92	0.75	0.002	0.87	0.87	0.76
Boosted classification trees	0.42	0.40	0.96	0.82	0.73, 0.89	0.75	0.06	0.76	0.82	0.68
Support vector machines	**0.65**	**0.64**	**0.96**	**0.88**	**0.80, 0.93**	**0.75**	**0.001**	**0.84**	**0.89**	**0.80**
C5	**0.57**	**0.60**	**0.93**	**0.85**	**0.76, 0.91**	**0.75**	**0.01**	**0.75**	**0.87**	**0.76**
Boosted logistic regression	0.59	0.55	0.97	0.87	0.78, 0.93	0.77	0.01	0.84	0.88	0.76
*Financial word lists—matched-pair set scenario*										
Stochastic gradient boosting	0.28	0.72	0.56	0.64	0.49, 0.77	0.5	0.03	0.62	0.66	0.64
Boosted classification trees	0.36	0.80	0.56	0.68	0.53, 0.80	0.5	0.007	0.64	0.73	0.68
Support vector machines	**0.4**	**0.76**	**0.64**	**0.70**	**0.55, 0.82**	**0.5**	**0.0033**	**0.67**	**0.72**	**0.70**
C5	**0.4**	**0.72**	**0.68**	**0.70**	**0.55, 0.82**	**0.5**	**0.0033**	**0.69**	**0.70**	**0.70**
Boosted logistic regression	0.12	0.60	0.52	0.56	0.41, 0.70	0.5	0.23	0.55	0.56	0.56

Bold values indicate best performing classifiers

A matched-pair set scenario with a reduced data set of 102 fraud and 102 non-fraud reports was also set up. The motivation was to gauge how the sensitivity measure changed in the unbalanced data set. It is somewhat adversely impacted as the classifiers designate more fraud cases as non-fraud. However, the balanced accuracy measure smooth out the differences. This is borne out by the balanced accuracy results for both scenarios which are analogous.

### Coh–Metrix Produces 110 Indices for Text Examined

However, once passed feature selection these indices were reduced to 29 having discriminating ability. Those indices shown in Table 2 overlap with previous findings in linguistic analysis to detect deception as illustrated in Table 1. For example, Coh–Metrix indices (prefixed by ‘DR’ and ‘SY’) indicate that there is a difference in the syntactic structure between fraud and non-fraud firms. This correlates with the view that deception in text is manifested by dense syntactic structure to reduce readability and comprehension.

There is also a difference in the use of adverbs and adjectives, and as indicated in Table 1, this can qualify the meaning of statements. Further, there is a difference in the use of connectives which can again lead to poor cohesion if used sparingly.

Referential cohesion measures (prefixed by ‘CR’) are also showing up as discriminators. This could again be the case that fraud firms are attempting to obfuscate the narratives through poor co-referencing.

To reinforce the view that deceiver’s use of linguistic constructs is distinct, *n*-gram language modelling was performed over the corpus. As Rutherford [58] argues for many words, patterns of word usage are stable in the financial domain as it constitutes an identifiable genre of text. However, where there are differences, significance can be attached to them. SVM and SGB show the best overall performance using metrics such as sensitivity, pos pred value and kappa as the main determinants of success. These values are important as they reveal the ability of the classifiers to detect a fraud firm. This ability is key in the unbalanced data set where non-fraud firms dominate. Further, the financial word lists also enabled a distinction between the fraud and non-fraud reports. This corresponds with the literature which indicates more negativity in fraud reports, greater use of modal words and passive verbs (see Table 1).

The decision tree classifiers (C5, Random Forest, SGB and CART) have performed well. They are known to filter out properties in the data that are insignificant. The resultant trees are often interpretable and can reveal what inputs are the best predictor of the output.

However, they are known to slide towards overfitting the training data, and this is true in cases where big data pushes the classifier to increase the splitting of the tree. Splitting can be reduced using a random forest technique and boosting. However, this can add complexity to the tree rendering it harder to understand. They are known to perform well only if a few highly relevant attributes exist. This indicates that this is likely the case in this corpus, given its moderate size and a reasonable number of attributes. This has enabled a successful discrimination between fraud and non-fraud reports.

Generally, SVMs are better at text classification given that it is marked by high dimensional space. This is due to their ability at identifying complex boundaries to separate the data. They are also more resistant to overfitting as they only need a core set of points, the support vectors which help to identify and set the boundary. They are ideal for this binary classification task, given its moderate corpus dimensions. This ability has been translated to results with a robust overall performance under the three approaches.

Logistic regression was the poorest performing classifier indicating that the reports are not linearly separable. This could perhaps be improved through feature engineering.

Each classifier has its strengths and weaknesses, not all patterns are learnt well by individual algorithms. A possible solution to this problem would be to train a group of classifiers on the same problem. This group are known as ensemble classifiers and are cooperatively trained on a data set in a supervised manner. Given the encouraging performance metrics, as shown in Tables 4, 5, 6 and 7, it is likely that once combined the classifiers could exceed this for each of the three approaches.

## Conclusion

Three observations prompted this study: the rising incidence of financial fraud, the explosion in unstructured text content and how language could be used to hide true intent. From a data perspective, quantitative analysis has had a monopoly in the model building process of tackling financial fraud including financial statement fraud, whereas the abundance of qualitative data produced has been left largely untapped. Language is a powerful tool upon which its users imprint their individual stamp. In this study, it was shown that an imprint such as deception is identifiable using linguistic constructs extractable using natural language processing tools.

Particularly, the measurement of financial text readability was advanced using Coh–Metrix. This is a robust NLP tool that examined the text more thoroughly for coherence and cohesion [18]. There is much that can be done to further work in this field of automated linguistic analysis for fraud detection. For example, an increase in the corpus size would better capture the language use in this genre and reinforce the validity of findings. Dimensionality reduction techniques such as latent semantic analysis as well as other feature selection techniques could be applied to determine if classification accuracy could be improved [6]. It has been demonstrated, however, that through judicious selection of linguistic features, firms that are possibly fraudulent could be marked out in an automated manner using machine learning technology. The approaches used are well suited for construction of an early warning information system to detect fraud.

Further use of natural language processing tools that pick up more subtle differences in sentiments in narratives could be used [20]. Other techniques such as clustering and vector space modelling using TF-IDF scores could also be executed over the corpus to determine success at discriminating a fraud from a non-fraud document [56]. It is hoped this preliminary study showed that the language used in financial narratives is revealing and can be deployed to aid in model building for fraud detection.
